# Molecular mechanism of azoles resistant *Candida albicans* in a patient with chronic mucocutaneous candidiasis

**DOI:** 10.1186/s12879-020-4856-8

**Published:** 2020-02-11

**Authors:** Ming-rui Zhang, Fei Zhao, Shuang Wang, Sha Lv, Yan Mou, Chun-li Yao, Ying Zhou, Fu-qiu Li

**Affiliations:** 1 Department of Dermatology, the Second Hospital of Jilin University, No. 218, Ziqiang street, Nanguan district, Changchun, 130000 China; 2 National Institute for Communicable Disease Control and Prevention, Chinese Center for Disease Control and Prevention, State Key Laboratory of Infectious Disease Prevention and Control, Beijing, China

**Keywords:** Chronic mucocutaneous candidiasis, STAT1, *Candida albicans*, Azoles resistance, Over-expression, Drug efflux

## Abstract

**Background:**

More and more azole-resistant strains emerged through the development of acquired resistance and an epidemiological shift towards inherently less susceptible species. The mechanisms of azoles resistance of *Candida albicans* is very complicated. In this study, we aim to investigate the mechanism of azole-resistant *C. albicans* isolated from the oral cavity of a patient with chronic mucocutaneous candidiasis (CMC).

**Case presentation:**

CMC diagnosis was given based on clinical manifestations, laboratory test findings and gene sequencing technique. Minimum inhibitory concentration (MIC) of the fungal isolate, obtained from oral cavity termed as CA-R, was obtained by in vitro anti-fungal drugs susceptibility test. To further investigate the resistant mechanisms, we verified the mutations of drug target genes (i.e. ERG11 and ERG3) by Sanger sequencing, and verified the over-expression of ERG11 and drug efflux genes (i.e. CDR1 and CDR2) by RT-PCR. A heterozygous mutation of c.1162A > G resulting in p.K388E was detected in STAT1 of the patient. The expression of CDR1 and CDR2 in CA-R was 4.28-fold and 5.25-fold higher than that of type strain SC5314, respectively.

**Conclusions:**

Up-regulation of CDR1 and CDR2 was mainly responsible for the resistance of CA-R. For CMC or other immunodeficiency patients, drug resistance monitoring is necessary.

## Background

Chronic mucocutaneous candidiasis (CMC) is a heterogeneous disease in children featured by persistent and recurrent infections of skin, nails and mucous membranes caused mainly by *Candida albicans* [1, 2]. The factors that predispose host to CMC infection could be autosomal or acquisitive. Increasing evidence indicates that some immunologic and hormonal abnormalities are associated with CMC due to changes of cellular immunity, which then lead to subsequent autoimmune endocrine disorders [3, 4]. To date, heterozygous gain-of-function (GOF) mutations of signal transducer and activator of transcription 1 (STAT1) have been identified as a cause of CMC [5–7]. Afterwards, many studies have proved that mutations at different sites of *STAT1* are associated with the pathogenesis of CMC [8–10].

Currently, azole anti-fungal drugs have been widely used for treating CMC as they show satisfactory bio-availability and safety. Unfortunately, there are an increasing number of resistant strains of *Candida spp.* in the presence of long-term azoles exposure. The potential mechanisms are associated with up-regulation of pharmaceutical transporters, over-expression or alteration of the drug target, as well as cellular changes caused, in some cases, by non-target effects induced by stress response [11].

In this study, we investigated the mechanism of azole-resistent *C. albicans* isolated from oral cavity in a CMC patient. We aim to remind the physicians that patients with congenital immunodeficiency should be monitored for the emergence of yeasts resistant to antifungals when they are on long-term medication. Also, it is necessary to pay attention to whether new drug resistance would be generated in immunodeficiency hosts.

## Case presentation

### Clinical information

A 15-year-old boy was admitted to the Dermatology Department of the Second Hospital of Jilin University due to recurrent thrush for more than 10 years. The patient was of full-term normal delivery, with non-consanguineous marriage of his parents. Family history revealed no members with relevant infectious diseases, genetic abnormalities or immunodeficiencies. During the long course, the patient had been taking oral azole antifungal drugs intermittently for many years and the specific dose was unknown.

### Clinical examination findings

**Fig 1:**
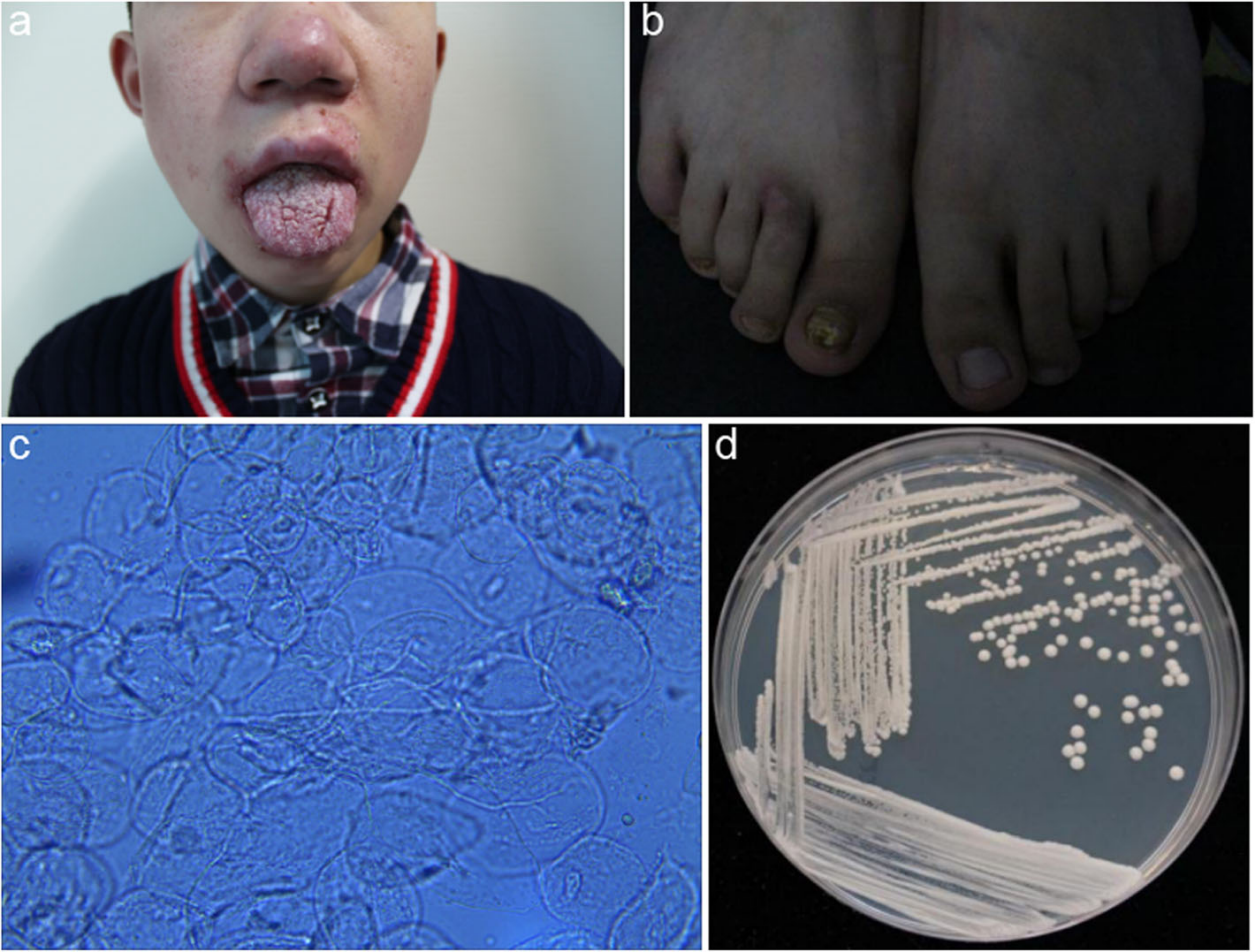
Clinical manifestations and mycological examinations of the patient. **a** Obvious white membrane was observed on the oral mucous membranes and tongue, which was cracked with pain. Erythema and papules were scattered on his face; **b** his right toenails was hyperkeratotic with brown discoloration; **c**, Direct microscopic examination of mucous samples revealed the presence of yeast cells along with pseudohyphae. **d**, Fungal culture reveals white creamy yeast colonies (SDA, 2 days at 28 °C)

There was obvious white membrane on his oral mucous membranes and tongue, which was cracked with pain. There were erythema and papules on his face (Fig. 1a). The right sided toenails were hyperkeratotic with brown discoloration for many years (Fig. 1b).

### Laboratory findings

Direct microscopic examination of mucous samples, which were obtained from patient’s oral cavity by sterile swabs, revealed the presence of yeast cells along with pseudohyphae, resembling *Candida spp.* infection (Fig. 1c). Typical white creamy yeast colonies were grown on Sabouraud Dextrose Agar (SDA, Fig. 1d). *C. albicans* was identified as the causative agent by sequencing of internal transcribed spacer region (ITS). Anti-fungal drugs susceptibility test showed that the minimun inhibitory concentration (MIC) of anidulafungin, micafungin, caspofungin, 5-flucytosine, posaconazole, voriconazole, itraconazole, fluconazole, and amphotericin B were 0.125 μg/m L, 0.06 μg/m L, 0.25 μg/m L, 0.125 μg/m L, > 8 μg/m L, > 8 μg/m L, > 16 μg/m L, > 256 μg/m, and 1 μg/m L. respectively. Lymphocytes analysis showed CD4+/CD8+ 1.02 (normal range 1.06–2.66), CD3-CD19+ 22.0% (normal range 5.0–18.0), CD3-CD(16 + 56) + 4.7% (normal range 7.0–26.0), other laboratory examinations, including complete blood count, serum immunoglobulin levels, and serum complement levels were normal. Glycemia, thyroid, parathyroid and adrenal hormones were within physiological limits.

### Diagnosis and treatment

On the basis of all the above findings, we made a diagnosis of CMC with resistant to azoles. However, after informing the patient and his family of the currently feasible treatments and considering the economic status of the patient’s family, the patient was still treated with itraconazole 200 mg (bid, PO). Fortunately, partial improvement was observed after 1 week. The patient is still under treatment now.

### Mutation in STAT1

**Fig 2:**
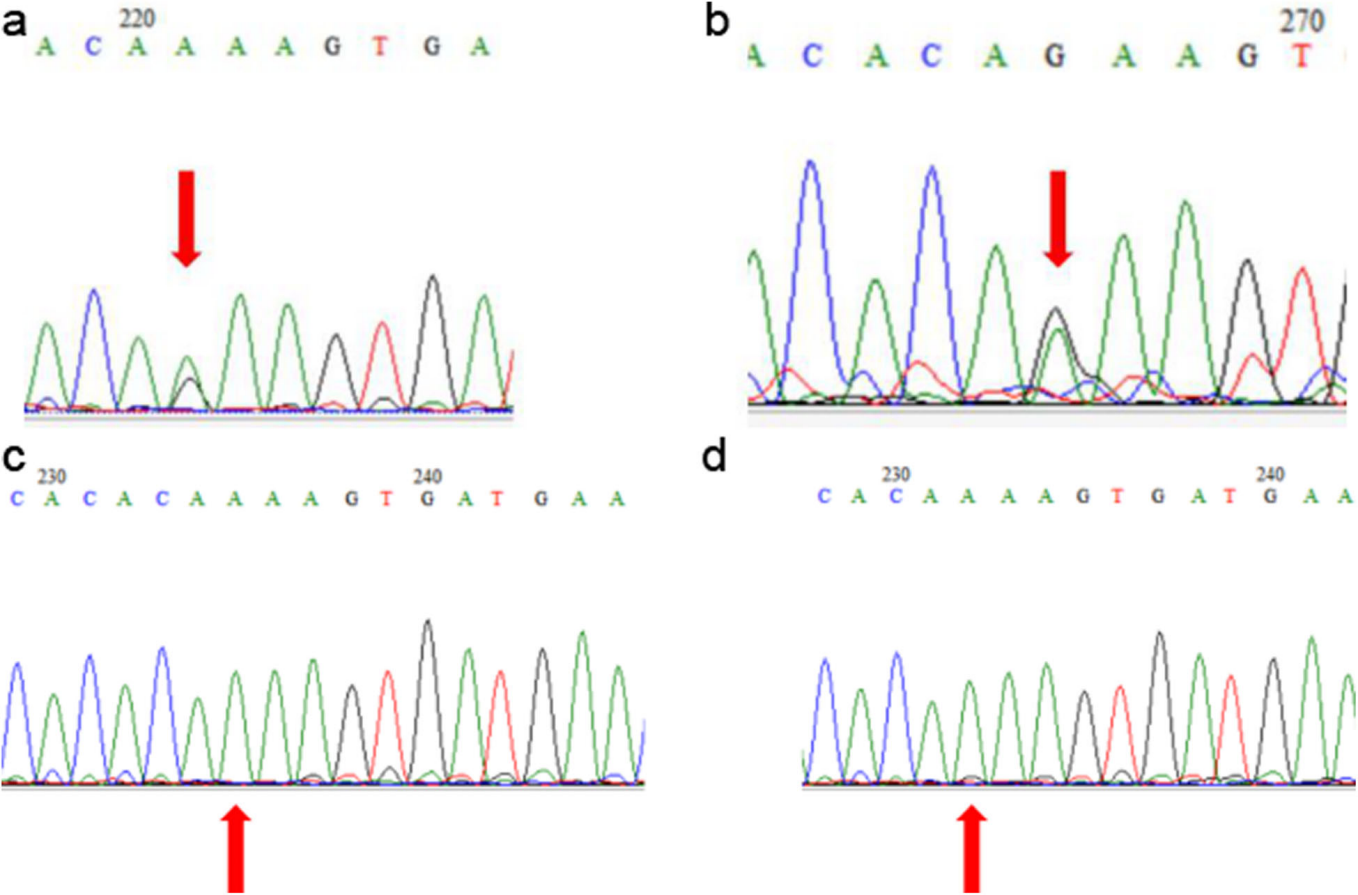
Direct sequencing analysis of *STAT1* exon 14 in patient and his parents. Forward (**a**) and reverse (**b**) sequence of the patient, forward sequence of the father(**c**) and mother(**d**) were shown. Patient had the heterozygous base change of c.1162A > G resulting in p.K388E in *STAT1*. The sequences of parents were normal

After signing the informed consent, peripheral blood was extracted by QIAamp DNA Blood Mini Kit (QIAGEN, Hilden, Germany). All exons of *STAT1* were amplified by PCR. Sequencing was performed by Sangon Biotech (Shanghai, China). A heterozygous mutation (c.1162A > G, p.K388E) was identified in exon 14 of the patient (Fig. 2a and b). Such mutation was not identified in the samples from his parents (Fig. 2c and d).

### ERG3 and ERG11 sequencing

*C. albicans* strain, termed as CA-R, was isolated from the oral cavity of the patient. The type strain SC5314 served as control. All strains were routinely cultured on yeast extract peptone dextrose medium (YPD) at 37 °C under constant stirring of 220 rpm/min for 12–24 h. *C. albicans* genomic DNA was extracted as described previously [12]. The extracted DNA was used as template for amplification of the full-length *ERG3* and *ERG11* genes, using the specific primers listed in Table 1. Compared with SC5314 strain, 8 nucleotide substitutions were identified in *ERG11* gene of CA-R and 6 nucleotide substitutions were identified in *ERG3*, respectively. Among these substitutions, 13 were synonymous mutations and 1 was missense mutations of ERG11 resulting in amino acid changes (p.E266D, Table 2). Amino acid exchange (E266D) was identified in fluconazole resistant isolates, however, such sequence differences may simply reflect the allelic variation.

**Table 1:** Primer sequences and annealing temperatures

Name	Sequences (5′-3′)	Annealing temperature(°C)
ERG3-F	GATCATAACTCAATATGG	60
ERG3-R	CTGAACACTGAATCG	
ERG11-F	ATGGATATCGTACTAGAA	60
ERG11-R	TCATTGTTCAACATATTC	
18S-F	TCTTTCTTGATTTTGTGGGTGG	58
18S-R	TCGATAGTCCCTCTAAGAAGTG	
CDR1-F	GCTGGTGAAGGTTTGAATGT	60
CDR1-R	CGCTGATGGTTGATGGATAG	
CDR2-F	ATCTGGTGCTGGTAAGAC	54
CDR2-R	GCTGATGGTTGATGGATAG

**Table 2:** Amino acid changes in ERG11 and ERG3 protein

Location		AA changes
ERG11	658	p.L220L
	798	p.E266D^a^
	996	p.V332V
	1026	p.K342K
	1110	p.L370L
	1203	p.Y401Y
	1296	p.A432A
	1302	p.A434A
ERG3	51	p.Y27Y
	306	p.T102T
	381	p.T127T
	402	p.Y134Y
	432	p.F144F
	438	p.F146F

^a^ reported to be unrelated to azole resistance of *C. albicans*

### Over-expression of drug efflux pump genes

RNA was isolated using RNeasy Mini Kit (QIAGEN, Hilden, Germany) according to the manufacturer’s instructions. The cDNA was synthesized with 1 μg RNA using the PrimeScriptTM RT Reagent Kit with gDNA Eraser (TaKaRa). RT-PCR was performed for independent amplification of internal reference gene (18S rRNA) and ERG11, CDR1, CDR2 genes to be quantified from the same cDNA template using a 7300 Real-Time PCR System (Applied Biosystems, Shanghai, China), with SYBR Premix Ex Taq (Tli RNaseH Plus. TaKaRa) using the primers listed in Table 1. PCR cycling conditions were 95 °C for 5 min, followed by 40 cycles of 95 °C for 5 s and appropriate annealing temperature for 30 s. SPSS 19.0 software was used for the analysis. The data were expressed as mean ± standard deviation (SD). The CT value of 18S rRNA was subtracted from the detected genes to obtain a ^Δ^CT value. The ^Δ^CT value of type material was subtracted from the ^Δ^CT value of CA-R to obtain ^ΔΔ^CT. The relative quantification was performed using the 2^-ΔΔCT^ method [13]. Expression of *ERG11*, *CDR1* and *CDR2* mRNA was detected in SC5314 and CA-R. The expression of *CDR1* mRNA in CA-R was about 4.28-fold than that of the SC5314, while the expression of *CDR2* mRNA in CA-R was about 5.25-fold than that of SC5314. The expression of *ERG11* mRNA in CA-R was about 0.79-fold than that of the SC5314 (Table 3).

**Table 3:** Drug efflux pump gene mRNA relative expression levels of CDR1, CDR2 and ERG11

	CDR1	CDR2	ERG11
^−^x ± SD(ΔΔCT)	−2.099 ± 0.189	−2.392 ± 0.478	0.348 ± 0.218
2^-ΔΔCT^	4.28	5.25	0.79

## Discussion and conclusions

*Candida* are normally non-pathogenic microorganisms in human beings. However, they are the major opportunistic fungal pathogens causing surface and invasive infections in individuals, which is associated with significant morbidity and mortality [9]. Immunosuppressive therapy, mucous damage, indwelling catheters, and prolonged hospital stay are the risk factors for *Candida* infection [14]. Besides, genetic factors must play a role in the pathogenesis of *Candida* infections, especially invasive candidiasis and CMC.

Several genes affecting the anti-fungal immunity have been reported to be associated with the pathogenesis of CMC, including *AIRE*, *CLEC7A*, *CARD9*, *IL17RA*, *IL17F*, *IL2Rα*, *Dectin-1*, *STAT1* and *STAT3* [5–7, 15–20]. *STAT1*, one of the seven transcription factors of the STAT family, is the major signaling components of interferon responses. In the presence of interferon binding to its receptor, *STAT1* is activated by tyrosine phosphorylation [21]. Upon forming a dimer, * STAT1* enters the nucleus and triggers the transcription of its targets. Such process plays a pivotal role in the defense against pathogens. The mutations of *STAT1* were considered as a GOF type because of a gain of phosphorylation and a loss of nuclear dephosphorylation. In a previous study, Van de Veerdonk et al. [6] demonstrated that patients with heterozygous missense mutations in the coiled-coil domain of *STAT1* had deficiencies in mounting TH1 and TH17 responses due to defective IL-12 receptor and IL-23 receptor signaling pathways [7]. S. Takezaki et al. [8] firstly reported that the GOF mutations of DNA-binding domain were also the genetic cause of CMC, which involved a gain of *STAT1* function due to impaired dephosphorylation in the coiled-coil domain mutations.

In this case, direct sequence analysis of *STAT1* exons indicated presence of c.1162A > G, which then resulted in p.K388E in exon 14. As previously described [10, 22], K388E was a GOF mutation in the DNA-binding domain. The patients showed an early onset and received different systemic or topical anti-fungal treatments for more than 10 years, especially azoles. Long-term azoles exposure may lead to generation of *C. albicans* resistant strains. Therefore, we speculated that the azoles-resistant *C. albicans* isolated from this case was induced by long-term exposure of anti-fungal drugs.

Azole drugs can target the ergosterol bio-synthetic pathway. Ergosterol is a crucial component of membrane of the fungal cell. Interruption of its synthesis allows accumulation of 14 α-methyl sterols, which is encoded by *ERG11* in *Candida spp.* The function of 14 α-methyl sterols is to alter the membrane stability, permeability, and the action of membrane-bound enzymes [23, 24]. Additionally, inhibition of ERG3, a Δ^5,6^-desaturase could lead to a depletion of ergosterol and accumulation of 14a-methylfecosterol, which allowed continuous growth in the presence of azole despite altered membrane composition [25]. Also, *ERG11* over-expression confers azole resistance, which is more common among azole resistant isolates of *C. albicans* [26]*.* Over-expression of efflux pumps, encoded by genes of the ATP-binding cassette (ABC) super-family or the major facilitator super-family (MFS), is the most common cause for drug resistance. Such process could decrease the intracellular drug concentration by increasing target abundance. Hence, more drugs are required to inhibit the activity of pathogens, which then results in reduction of drug susceptibility [27].

To further investigate the resistance mechanism of *C. albicans* in this case, we sequenced *ERG11* and *ERG3*. In total, 14 nucleotide substitutions were detected in *ERG11* and *ERG3*, among which 13 nucleotide substitutions were synonymous mutations and 1 was missense mutations of *ERG11* resulted in amino acid changes (p.E266D). In the previous study, such mutation was not reported to be related to azole resistance in *C. albicans* [28]. Additionally, RT-PCR was performed to detect the expression of *CDR1*, *CDR2* and *ERG11* mRNA between CA-R and the type strain SC5314. The relative expression of *CDR1* and *CDR2* rather than ERG11 in multiple azole resistant strains was obviously higher than that of SC5314 strain. These suggested that the main cause for azoles-resistant CA-R may be related to the over-expression of CDR1 and CDR2.

Several aspects have been reported to affect the expression of the azole target and/or drug pumps, including mutations in the drug target enzyme and efflux pumps over-expression, loss of heterozygosity, increased chromosomal copy number, aneuploidy, as well as the isochromosome [29]. These constituted the main reasons of azole resistance of *C. albicans* together with the biofilm formation [30]*.* They can occur in a single set or concurrently, which can produce additive effects or lead to cross-resistance among azoles. In this study, we only focused on the determination of drug efflux pump genes and target enzyme sequence. In future, additional studies are required to investigate whether there are other causes of drug resistance.

With the increasing understanding on CMC mutation mechanism, strategies based on the defects in *STAT1* GOF mutations may serve as a candidate for treating CMC, such as decreasing hyperphosphorylation of *STAT1* and restoring Th17 function by blocking inhibitory mechanisms [31]. However, the efficacy of these treatment options has not been well confirmed. Therefore, long-term systemic application of anti-fungal drugs is often preferred in clinical settings. Clinically, within a safe range, these patients were usually given a higher therapeutic dose of the drugs. After the symptoms were controlled, it was gradually reduced and finally the maintenance treatment was performed at the smallest controllable dose. Drug exposure in the form of prophylaxis, repeated, or long-term therapy is associated with the emergence of resistance [30]. At present, there are no system reports about anti-fungal drugs susceptibility of pathogen in CMC patients, who were receiving long-term oral anti-fungal treatment. Therefore, further investigations are still needed. Over-expression of the efflux pump was mainly responsible for the drug resistance. Efflux pump over-expression will reduces the entry of the drug into the pathogens, thereby reducing the effect of the drug. However, in presence of increased extracellular drug concentration, it may still be effective [30]. Although the pathogen in this case is resistant to the multiple azoles, the anti-fungal drug is still selected after informing the patient and his family of the currently feasible treatment options. Fortunately, the symptoms showed remission after administration of itraconazole in this case.

There are some limitations in this study. Firstly, there was only one case. Although the number of studies on gene mutation induced CMC showed increase, there are still rare cases in clinical settings. Secondly, we only investigated the over-expression of target genes encoding the enzyme and the mutation of target genes. We did not investigate the roles of other drug-resistant mechanisms.

In conclusion, we reported a case of CMC with multiple azole-resistant *C. albicans*, and investigated the possible mechanism of the drug resistance. The expression of *CDR1* and *CDR2* gene was up-regulated. For special patients, such as CMC or other immunodeficiency patients, drug resistance monitoring is very necessary.

## Data Availability

The datasets used and/or analyzed during the current study are available from the corresponding author on reasonable request.

## Abbreviations

ABC: ATP-binding cassette superfamily
CMC: Chronic mucocutaneous candidiasis
CT: Cycle threshold
GOF: Gain-of-function
ITS: Internal transcribed spacer region
MFS: Major facilitator superfamily
MIC: Minimun inhibitory concentration
PCR: Polymerase chain reaction
qRT-PCR: Quantitative real-time reverse transcription PCR
SDA: Sabouraud Dextrose Agar
STAT1: Signal transducer and activator of transcription 1
